# Community participation and operational lessons from implementing a protective coating as a novel vector control tool in dengue-endemic neighborhoods of Cúcuta and Los Patios, Colombia

**DOI:** 10.1186/s12889-026-29272-2

**Published:** 2026-09-01

**Authors:** Susana Koo Chong, María Angelica Carrillo, Rocío Cárdenas Sánchez, Tatiana Rivera-Ramirez, Axel Kroeger

**Affiliations:** 1 Centre for Medicine and Society/ Planetary Health, Albert-Ludwigs University Freiburg, Freiburg in Breisgau, Germany; 2 Research Group GIGA, University Francisco de Paula Santander, Cucuta, Colombia

**Keywords:** Dengue, Vector control, Protective coating, Community participation, Novel vector control tool, Lessons learned

## Abstract

**Background:**

The control of Aedes-borne diseases (dengue, Zika, chikungunya, yellow fever) is a high public health priority in tropical and subtropical countries. The adoption of new vector control tools, such as protective coating (PC), requires understanding the social dynamics that influence their acceptance and feasibility. This study investigated the experiences and perspectives of diverse stakeholders involved in a vector control project in urban Colombia, with the aim of assessing community acceptance and operational feasibility. Insights gained are important for optimizing implementation and gauging the broader viability of innovative vector control strategies.

**Methods:**

A qualitative descriptive study was conducted using focus group discussions and in-depth interviews with 70 key stakeholders, including community leaders (*n* = 3), community volunteers (*n* = 30), vector control staff (*n* = 7), supervisors (*n* = 5), and community residents (*n* = 25). Data was collected after the intervention and analyzed using inductive thematic content analysis with open coding. Participation was voluntary and confidential.

**Results:**

The protective coating was perceived as feasible and generally well accepted by the community. Facilitating factors included the involvement of community leaders, strong volunteer commitment, and effective coordination among institutions. Challenges such as trust issues, logistical constraints, and safety concerns were identified and addressed through adaptive strategies, including adjusted work schedules and strengthened communication.

**Conclusion:**

Community involvement, effective communication, and adaptive management were key to the successful implementation of the protective coating and helped address logistical challenges. The lessons learned provide useful information for scaling up novel vector control approaches in similar urban settings in Latin America.

**Supplementary Information:**

The online version contains supplementary material available at https://doi.org/10.1186/s12889-026-29272-2.

## Background

Dengue fever, a vector-borne viral disease, is a global public health concern, particularly in tropical and subtropical countries [1–3]. Transmitted primarily by day-biting *Aedes* mosquitoes, this disease represents a public health threat to about half of the world population [3, 4]. Current global estimates indicate 100–400 million infections annually [5, 6]. Clinical presentations range from mild flu-like to severe clinical forms that may lead to shock and death if not treated [3, 6]. The burden associated with dengue involves not only the health impact but also entails high economic and social costs for the affected communities and healthcare systems [7–9].

In Colombia, dengue remains a persistent public health challenge, and the country is highly endemic for arboviral diseases such as dengue, Zika, and chikungunya, all transmitted by *Aedes* mosquitoes. The country experiences regular outbreaks, with incidence between 2012 and 2020 ranging from 90.7 to a maximum of 476.2 cases per 100,000 inhabitants [10–12]. The burden varies across regions [13–15], influenced by factors such as levels of urbanization, socioeconomic disparities, and unequal access to healthcare [16, 17].

Understanding these specific challenges within Colombia is crucial for developing effective interventions [18, 19]. In addition to these structural factors, efforts to reduce the number of dengue cases in the past have been hindered by a series of factors, including rapid urbanization, increased travel and commerce, climate change and inadequate domestic water management, and poor vector control [20–23]. These conditions create a favorable environment for mosquito breeding, facilitate the transmission of arboviral diseases [24, 25], and undermine the effectiveness of standard vector control methods [26].

Conventional vector control methods, such as environmental management, insecticide spraying and larvicide application in domestic water containers, have shown limited effectiveness due to resource constraints, short residual activity of available products and operational challenges. Increased involvement of local communities in control operations has been proposed as a key component for improving vector control outcomes [27, 28].

One of the innovations in this field is the use of protective coating (PC) applied to domestic and peridomestic water containers. Carrillo et al. [29] reported that laundry tanks accounted for more than 90% of all mosquito pupae in the two study cities (Cúcuta and Los Patios). The intervention therefore consisted of the application of a slow-release product with a dual effect: (a) killing adult mosquitoes resting on the tank walls using a pyrethroid insecticide (alphacypermethrin) and (b) inhibiting larval development using an insect growth inhibitor (pyriproxyfen). The PC is based on a physicochemical process whose main characteristic lies in the creation of polymeric microcapsules, which encapsulate the two active ingredients (commercial name SATIS by INESFLY company in Spain). Before large-scale implementation, laboratory and semi-field trials were conducted to assess its efficacy and safety [30].

The protective coating was subsequently evaluated in a cluster-randomized trial conducted in 20 clusters in Cúcuta and Los Patios, Colombia, as reported in Cárdenas et al. [31, 32]. The trial was informed by a baseline study and involved the application of the coating in intervention clusters, while control clusters received routine vector control activities. Local health authorities and community volunteers collaborated closely in the implementation of the intervention. This manuscript presents the qualitative findings from focus group discussions and interviews conducted during the implementation of the protective coating intervention. It explores the experiences and perceptions of key stakeholders and identifies operational and community-related factors that influenced acceptance and feasibility. These qualitative results complement the cluster-randomized trial reported elsewhere [31, 32].

## Methods

### Study area

Cucuta, the capital of the North of Santander State, is the main urban center of the Metropolitan Area of Cúcuta. Located at 7° 54’ 27” N / 72° 30’ 17” W [33], it has an average altitude of 320 m and covers 1,176 km², representing 5.65% of the Norte de Santander department. The population increased from 711,715 inhabitants in 2018 (DANE census) to 777,106 in 2020 [34], with 93.4% living in the urban area. This strong urbanization results in a population density of 12,708 inhabitants per km² in urban zones and 20.5 inhabitants per km² in rural zones [35]. The climate is characterized by a mean temperature of 27.6 °C and an annual precipitation of 806 mm [36].

Los Patios, located 7 km from Cúcuta at 7° 50′ 17″ N / 72° 50′ 47″ W, has an average altitude of 410 m and covers 131 km² [37]. It has a tropical climate with an annual mean temperature of 27 °C [38]. In 2025, the municipality had 104,287 inhabitants, of whom 101,782 lived in the urban area, with a density of 603.27 inhabitants per km² [39]. Due to the high prevalence of dengue in its urban neighborhoods, the qualitative study focused on this municipality.

### Community involvement

Community participation was central to the implementation of the protective coating intervention. Local health authorities collaborated with municipal vector control departments in Cucuta and Los Patios, community leaders, and volunteers throughout the project. Community leaders were given detailed information on the purpose of the project and the phases in which it would be developed: promotional campaigns, implementation (which included the application of PC in the laundry tanks), and final evaluation of the project [31, 32].

**Fig. 1:**
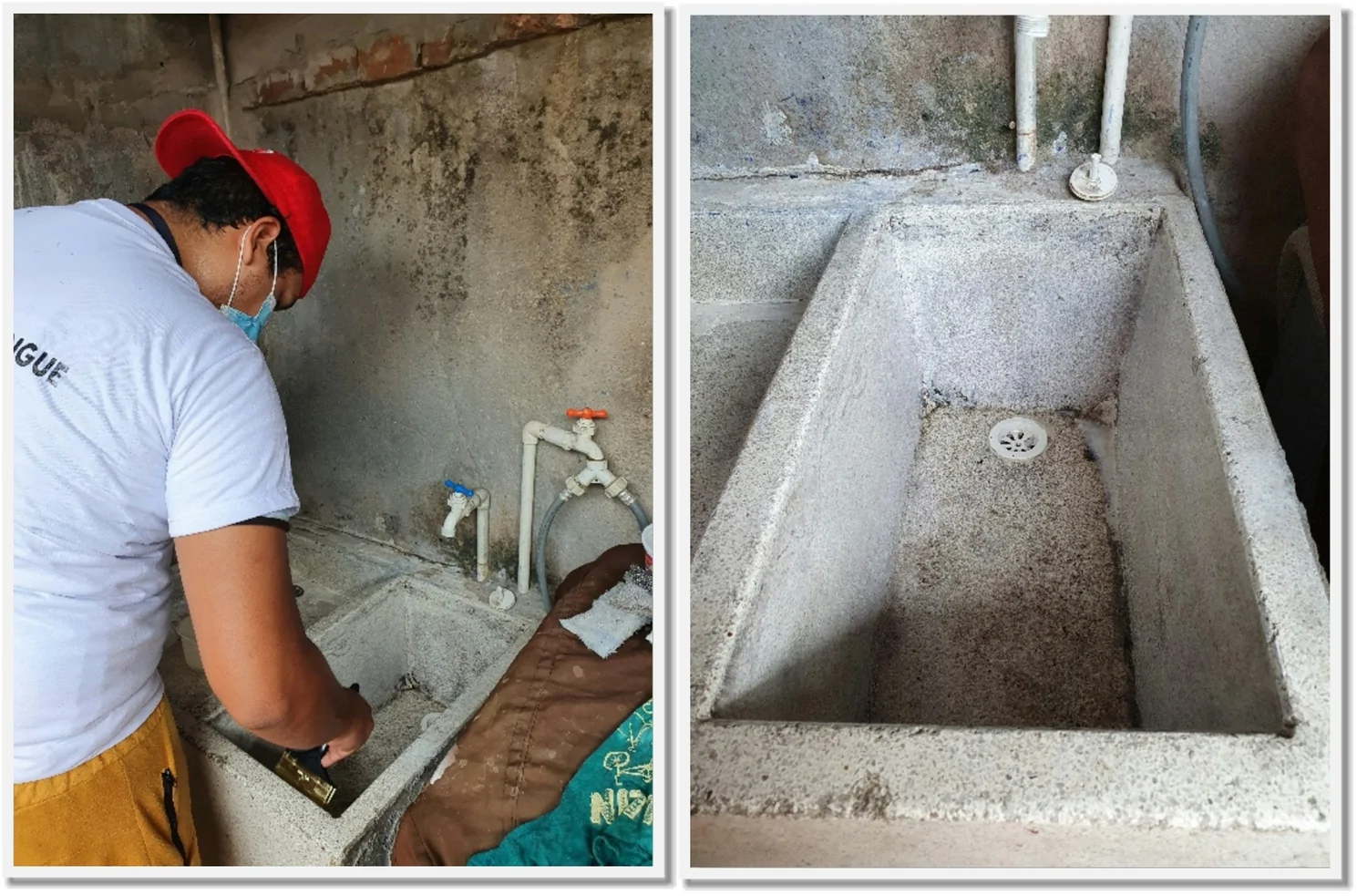
Application of the coating product in domestic water containers. The slightly white color disappears after drying

Community leaders helped introduce the intervention in their neighborhoods, and volunteers supported promotional activities and the application of the protective coating. The project also promoted gender equality by allowing adults (> 18 years) to choose their preferred role (either “promoters” or “painters”) based on comfort and skills, challenging traditional norms around manual labor [31, 32]. Additional operational details related to volunteer roles, scheduling, household preparation, and fieldwork procedures are provided in Appendix A . (Fig. 1).

### Participants selection for the qualitative study

The qualitative study was conducted at the end of the cluster randomized trial, after the protective coating intervention had been fully implemented in the intervention clusters. To capture diverse perspectives and perceptions on the new vector control tool, stakeholders actively involved in the project were invited to participate in focus group discussions (FGDs). The stakeholders included: (a) municipal vector control technicians (staff from the department of vector control), (b) community leaders, (c) community volunteers (promoters, painters, and supervisors), and (d) community residents who received the intervention.

A total of 13 FGDs were carried out, each with 5–10 participants. The FGDs were stratified by stakeholder type to ensure participants felt comfortable expressing their views and to capture diverse perspectives. Separate FGDs were conducted with municipal vector control technicians, community leaders, community volunteers (promoters, painters, supervisors), and community residents who received the intervention. Each group discussed trust, communication, operational challenges, safety, and perceptions of the protective coating.

Participation was voluntary, and invitations to participate were sent via WhatsApp messages and verbal communication. In addition, five in-depth interviews were conducted: three with community leaders and two with municipal vector control technicians. Details of participant characteristics are presented in Table 1.

**Table 1:** Overview of participants in the qualitative study by role and municipality/neighborhood

Role	Municipality/Neighborhood	Interviews	No. of respondents
1. Community presidents and leaders	Cucuta/ Aeropuerto Cucuta/ Atalaya Cucuta/ San Martin	3 (in depth interviews)	3
2. Community volunteers	Cucuta/ Comuneros Cucuta/ El Trigal Los Patios/Once de Noviembree and Videlso Cucuta/ San Martin Cucuta/ San Luis	5 FGDs	30
3. Municipal vector control technician	Cucuta and Los Patios municipality	2 FGDs	7
4. Supervisor	Cucuta/ Aeropuerto Cucuta/ Comuneros Cucuta/ San Luis Cucuta/San Martin Los Patios/Videlso Los Patios/Patio Centro	1 FGDs	5
5. Community resident	Cucuta/ Atalaya Cucuta/ Comuneros Cucuta/ El Trigal Cucuta/ San Martin Cúcuta/ Ospina Pérez	5 FGDs	25

### Data collection

The 13 FGDs (with a total of 67 participants) and 3 in-depth interviews were audio-recorded, and manual notes were taken to capture useful context for later analysis, with prior authorization from the participants. Interviews were conducted in Spanish by the principal investigator using a previously established list of guiding questions (see Supplementary File 1) to maintain consistency in the interviews, ensure that topics of relevance were addressed, and to better understand the experiences and perspectives of the participants according to their role. Each interview lasted 40 to 60 min and was conducted in familiar settings for participants (community centers, homes, private rooms in a coffee shop), to promote comfort and to avoid possible bias in their responses. Participation in FGDs was confidential, and all transcripts were anonymized during analysis. No names or identifying information were included in the research results.

### Data analysis

A qualitative content analysis (QCA) procedure was used to identify themes related to the experiences and viewpoints of participants through a process of coding as well as the recognition of patterns. This procedure was based on the QCA model [40], an inductive approach where themes and categories are developed from the data.

The audio-recorded interviews were manually transcribed into Microsoft Word. To gain a rich comprehension of the data, as well as identify trends, patterns, and concepts within the conversation, the transcribed text was read several times. The transcribed text was read repeatedly to gain an overall understanding of the data. Meaning units were defined as segments of text (words, sentences, or paragraphs) that expressed a single idea relevant to the research question.

These meaning units were then condensed to preserve their core meaning while reducing unnecessary detail. Condensed units were assigned codes, representing the central concept in each segment. Codes were subsequently grouped into categories based on similarity in content. Categories reflected the manifest content of the data. Finally, categories were compared and merged into broader themes, representing the latent content and providing an interpretive understanding of the promoting and obstructing factors for the implementation of the protective coating.

Once the analysis of the transcripts was complete, findings were translated into English, maintaining the original meaning as well as the semantic consistency of local expressions and context. Results were summarized in a written narrative and supported with participants’ quotes to help explain the results and share their experiences.

### Ethical considerations

The study was conducted following ethical standards and principles, ensuring the transparency and integrity of the research. Verbal informed consent was obtained from community members, and written consent was obtained from all participants in the qualitative study. Details of the study objectives and procedures were provided, assuring participants that their participation was voluntary and without risk, and that they could withdraw at any time without consequences. Participants’ privacy was ensured, and participation in the focus group discussions was confidential rather than anonymous. Although participants knew each other within the group, all transcripts were anonymized during analysis, and no identifying information is reported in the results. The data collected were securely stored and deleted six months after the conclusion of the project, following confidentiality and data protection regulations. The study obtained ethical approval from the Ethical Review Committee of Albert-Ludwigs Universität Freiburg, Germany, and was endorsed by the local health authorities in Colombia.

## Results

 The qualitative findings reflect the experiences and perceptions of community leaders, volunteers, supervisors, municipal vector control technicians, and residents involved in the implementation of the protective coating (PC). Four overarching themes emerged: trust and social dynamics, communication and community engagement, operational challenges influencing feasibility, and perceived usefulness and acceptance of the protective coating.

1. Trust-building dynamics shaped community acceptance of the intervention

Trust was central to community acceptance of the intervention. Community leaders emphasized their role as the primary communication bridge, noting that households often relied on their endorsement before agreeing to participate. Their involvement facilitated social mobilization and strengthened confidence in the project.

“*The community leader (president) is the one who communicates and informs the community. Sometimes the community asks if the community president gave his authorization; they feel more confident to participate*.” (Community leader, Atalaya/Cúcuta).

Leaders also highlighted the importance of selecting volunteers from within the same neighborhood. Familiarity with local dynamics and residents helped reduce mistrust and encouraged households to listen to the information provided.

“*The participation of community members as project volunteers was essential*,* as they have a deep knowledge of their neighborhood and its situation*.” (Community leader, San Martin/Cúcuta).

Volunteers described the initial barrier of approaching households and the need to “break mistrust” during the first contact. Their presence as neighbors helped establish rapport and facilitated conversations about dengue prevention.

“*I believe that the volunteer was a key element because they had to break mistrust when reaching out to the community*.” (Community volunteer, El Trigal).

However, distrust also emerged due to previous experiences of fraud in the neighborhoods. Some volunteers were rejected or treated with suspicion, particularly male volunteers visiting households where women were alone. To avoid misunderstandings, volunteers often worked in mixed-gender pairs.

“*There was a controversial issue because when the households saw my male work partner*,* they were afraid because he is a man and he is tall*,* so they were intimidated*.” (Community volunteer, San Luis).

In addition to trust-related dynamics, volunteers also described what motivated them to participate in the project. Some had previous experience in health campaigns, which encouraged them to join again. Others noted that their participation was driven by both the social benefits for their community and the small economic incentive provided as gratitude for their collaboration.

*“I was motivated to work on this project because I wanted to provide a social service to the community. Also to prevent the problems of dengue fever and so that all the neighbors could have access to paint.”* (Community volunteer, El Trigal).

Municipal vector control technicians played a crucial role in reinforcing trust. Their uniforms and long-standing presence in dengue campaigns made them recognizable and credible to residents.

“*Our uniforms work as identifiers*,* marking us as public health workers affiliated with the Departmental Health Institute*.” (Municipal vector control technician, Los Patios).

Supervisors observed that acceptance varied across neighborhoods. In socioeconomically disadvantaged areas, volunteers sometimes face rejection, insults, or accusations of having ulterior motives. In these situations, supervisors accompanied volunteers to clarify the purpose of the visits and reassure residents.

“*In this neighborhood*,* people did not accept the promoter*,* but when they saw the supervisor*,* their tone of voice changed*.” (Supervisor, El Aeropuerto).

These social dynamics shaped how households perceived the intervention and influenced the overall feasibility of the project.

2. Communication strategies determined the level of community engagement and clarity of project goals

A wide range of communication strategies was used to reach households and ensure awareness of the intervention. Personalized home visits were the primary method, complemented by loudspeaker announcements, church messages, WhatsApp broadcasts, and word-of-mouth communication.

Volunteers and leaders emphasized the effectiveness of combining formal and informal channels. Loudspeaker announcements before and during the campaign helped prepare residents for upcoming visits.

“*It is effective to make the broadcasting with loudspeakers several days before*,* and during the work week*.” (Supervisor, San Luis).

Church announcements also played an important role, leveraging the trust residents placed in religious leaders.

“*Churches are a good source of information; people go and listen to everything the priest says and of course that is it*.” (Community volunteer, El Trigal).

Community leaders used WhatsApp groups extensively to share information, photos of volunteers, and reminders about upcoming visits.

“*I informed the community through WhatsApp groups*,* and I called some people privately*.” (Community leader, Aeropuerto/Cúcuta).

Word-of-mouth communication between neighbors further strengthened acceptance. As households began to participate, others followed based on their neighbors’ experiences.

“*From the second day on*,* the percentage of people who said ‘no’ decreased*,* because someone started to spread the word*.” (Community volunteer, Los Patios).

Communication challenges also emerged. Some volunteers struggled to explain the purpose of the intervention clearly, especially those with limited formal education. Municipal technicians often had to revisit households to clarify misconceptions.

“*Some volunteers did not deliver an important message of the purpose of the project*,* which caused confusion*.” (Municipal vector control technician, Los Patios).

Technicians emphasized the importance of using simple language, maintaining a polite attitude, and adapting communication to local customs.

“*In a community with varying levels of education*,* the use of simple language becomes imperative*.” (Municipal vector control technician, Cúcuta).

These experiences underscored the need for strong communication skills, adequate training, and culturally appropriate messaging to ensure community engagement.

3. Operational and logistical challenges influenced the feasibility of implementing the protective coating

Although operational procedures are detailed in Appendix A and B, participants’ perceptions revealed how logistical factors shaped the implementation experience.

Morning hours were generally preferred due to cooler temperatures and higher household availability. However, in neighborhoods with predominantly young working families, houses were often closed during morning visits.

“*We arrived at the homes; the houses were closed*,* everyone was working*,* and the children were in school studying. The morning schedule was terrible*,* terrible*.” (Community volunteer, El Trigal).

Water shortages also affected feasibility. When water service was interrupted, households were unable to empty their laundry tanks, delaying the application of the protective coating.

“*They told us: how am I going to empty my tank*,* we just filled the tank after a week of not having water*.” (Community volunteer, San Luis).

Rain further disrupted activities, reducing household receptivity and forcing volunteers to pause work.

“*The rain would make people close their house and lock themselves in*.” (Community volunteer, El Trigal).

Supervisors monitored fieldwork quality and addressed inconsistencies in documentation. (see Appendix B for fieldwork tools and documentation procedures)They noted cases where volunteers did not inspect tanks properly or recorded incorrect information, which affected painters’ ability to locate households.

“*Although the promoters communicated the project information*,* there was some neglect in the tank inspections*.” (Municipal vector control technician, Los Patios).

Safety concerns also emerged. Two incidents, an armed robbery and an explosive device reported near one neighborhood, led to temporary suspension of activities.

“*The team experienced a security issue in the neighborhood*,* as three members were victims of an armed robbery*.” (Supervisor, Los Patios).

These challenges highlighted the importance of flexible scheduling, accurate documentation, and safety precautions during fieldwork.

4. Stakeholders perceived the protective coating as useful, acceptable, and beneficial for dengue prevention

Across all stakeholder groups, the protective coating was widely perceived as effective, safe, and beneficial. Municipal vector control technicians compared it favorably to conventional larvicides such as Dimilin and VectoBac^®^, noting its longer residual effect and lack of harmful impact on water.

“*The coating of the laundry tank lasts longer than Dimilin*.” (Municipal vector control technician, Cúcuta).

Technicians also reported personal observations of reduced mosquito larvae after applying the coating in their own homes.

“*When it was applied in my house*,* I never had larvae in that tank again*.” (Municipal vector control technician, Los Patios).

The heads of the household expressed their satisfaction with the effectiveness of the protective coating, claiming that it met their expectations, both based on the information provided about the product and based on their own observations (reduction in mosquito bites and absence of larvae in the laundry tanks).

“*For example*,* I could not comfortably sit at the table with my feet underneath and assist my children with their homework. This was because I often felt the sensation of mosquitoes landing on my legs*,* disrupting our activities*”. (Community member from Comuneros).

Residents appreciated that the coating did not alter the color, taste, or smell of water once dry, and that it did not leave residues, as do the commercial products (Dimilin and VectoBac^®^ applied by the vector control services. In addition, no adverse reactions were reported by users upon contact with the treated water. These aspects contributed significantly to the positive acceptance of protective coating.

“*I liked that they applied the paint in the laundry tank. When I saw that the paint did not produce any slime*,* nor did it produce any smell*,* nor any weird taste after applying it*,* I thought this product is 100% better than the other methods*”. (Community member from El Trigal).

Compared to chlorine tablets, residents noted that the coating was safer and did not bleach clothes or cause unpleasant tastes.

“*With this paint applied*,* we have the confidence to wash our face*,* wash our clothes*,* and even use that water to shower. Unlike when we use chlorine tablets*,* where it is not safe to brush our teeth because the water will have a chlorine taste*.” (Community member, Ospina Pérez).

They also valued the extended protection period of 6–9 months, which reduced the need for frequent reapplication of other products.

“ *It lasts many months*,* so we don’t have to apply products all the time*.” (Community member, El Trigal).

In addition, community members expressed keen interest in buying the protective coating for future use. Users estimated the product’s value at 10,000 to 20,000 Colombian Pesos ($2.00 to $4.00 US dollars) for 100–150 ml of paint, a quantity sufficient to cover the inside walls of the laundry tank. Notably, users considered the need for reapplication every 6 to 9 months in their cost estimation. This interest in investing in the product underscores the perceived value and efficacy of the protective coating within the community. Their willingness to purchase the coating reflects a strong sense of trust in the tool and suggests that its use could be sustained beyond the project’s implementation.

“*Having the experience and knowing that I have already used it and found it favorable*,* I would buy it*,* of course*,* at a reasonable price depending on my finances.”* (Community member, Comuneros).

Overall, the protective coating was viewed as a promising and acceptable vector control tool that addressed both practical and safety concerns.

## Discussion

The findings of this study highlight the social and operational factors that influenced the implementation of the protective coating (PC) in dengue-endemic neighborhoods of Cucuta and Los Patios. Trust, communication, and collaboration with local stakeholders were central to community acceptance, while logistical constraints and safety concerns shaped the feasibility of field activities.

Volunteers played a central role in promoting and applying the product. After receiving approval from community leaders, they became the first point of contact with households, helping to build trust and a positive perception of the intervention [41]. Community leaders emphasized the importance of communicating through them, as they serve as trusted intermediaries and help mobilize residents. This aligns with findings from Marubu [42] and Seale et al. [43], who describe the influence of community leaders in strengthening credibility and facilitating engagement in public health initiatives.

The results reaffirm the importance of community involvement and of engaging diverse stakeholders, including volunteers, health professionals, and government authorities, in achieving effective and sustainable management practices [44]. Active participation fosters a sense of ownership and responsibility, which has been described as essential for community-based programs [45]. In this study, residents perceived the PC as effective, noting the absence of mosquito larvae in their laundry tanks and expressing interest in using the product again. Clear communication about the benefits of the PC contributed to this acceptance, consistent with findings from Awasthi et al. [46] and Rivera et al. [47], who highlight the role of community engagement in sustaining vector control strategies.

Collaboration between supervisors and municipal vector control technicians was also important. Supervisors ensured the quality of fieldwork and provided support to volunteers, reinforcing teamwork and coordination [48]. Municipal technicians contributed technical expertise and credibility, helping to clarify misconceptions and strengthen trust among residents.

Several challenges were encountered during implementation. Logistical difficulties, communication barriers with household heads, and community reluctance were common. Similar barriers have been reported in dengue control programs in Vietnam and the Indo-Pacific region [49, 50]. These challenges underscore the need for adaptive strategies and risk management protocols to maintain project effectiveness [51].

Safety concerns also emerged during household visits. Volunteers sometimes felt unsafe or unwelcome, reflecting broader security issues in some neighborhoods. Gunn et al. [52] and Mathur et al. [53] describe comparable experiences in community-based vector control efforts, where field teams faced risks and hostile conditions. Gender-related dynamics also influenced household receptivity, particularly when male volunteers visited homes where women were alone. Studies by Mungall-Baldwin [54] and Gunn et al. [52] emphasize the importance of addressing gender considerations to support inclusive participation in vector control programs.

Logistical problems related to water availability affected implementation. Water shortages prevented households from emptying their laundry tanks, and irregular municipal water supply disrupted planned schedules. These issues are common in low-income neighborhoods and have been noted in other dengue control efforts [8]. Weather conditions also affected field activities, requiring adjustments such as rescheduling visits or encouraging households to find alternative ways to store water. Similar challenges have been documented by Espinoza-Gómez et al. [55], who highlight the need for flexible planning in response to climatic and infrastructural constraints.

Overall, the findings of this study emphasize the importance of trust, communication, and collaboration for successful community-based vector control interventions, while also illustrating the operational challenges that must be considered when implementing new tools such as the protective coating.

### Conclusions and recommendations for future community involvement programs

The implementation of the protective coating in laundry tanks was feasible and well accepted by community members in Cúcuta and Los Patios. Trust in community leaders, clear communication, and the active participation of volunteers were central to achieving household acceptance and ensuring correct application of the product. Operational challenges, such as water shortages, safety concerns, and scheduling constraints, highlight the need for flexible planning and strong coordination among local institutions.

Recommendations for future community-based vector control interventions.

1. Strengthening collaboration with local health authorities to ensure regulatory compliance, reinforce community trust, and facilitate access to technical and logistical resources.
2. Prioritize communication through community leaders, who serve as trusted intermediaries and help mobilize residents and clarify project objectives.
3. Provide continuous training for volunteers and field teams focused on communication skills, household engagement, and correct application procedures.
4. Implement simple monitoring and evaluation systems to track field activities, identify operational gaps, and support timely corrective actions.

Future research should assess the long-term sustainability, cost considerations, and scalability of protective coating interventions in other urban settings.

## Supplementary Information


Supplementary Material 1.



Supplementary Material 2.



Supplementary Material 3.


## Data Availability

The datasets generated and analyzed during the current study are not publicly available due to confidentiality and the risk of identifying participants but are available from the corresponding author on reasonable request.

## Abbreviations

DANE: National Administrative Department of Statistics (Departamento Administrativo Nacional de Estadística)
DFG: German Research Foundation
DSCV: Digital Surveillance of Vector Control
FGDs: Focus Group Discussions
IDEAM: Institute of Hydrology, Meteorology and Environmental Studies (Instituto de Hidrología, Meteorología y Estudios Ambientales)
IDS: State Health Institute (Instituto Departamental de Salud)
IVM: Integrated Vector Management
PAHO: Pan American Health Organization
PC: Protective coating
SATIS: The commercial name for the insecticidal coating product used in the study
WHO: World Health Organization
