# Supplementary material for: Community participation and operational lessons from implementing a protective coating as a novel vector control tool in dengue-endemic neighborhoods of Cúcuta and Los Patios, Colombia
Source: BMC Public Health. 2026 Sep 1;26:2505. doi: 10.1186/s12889-026-29272-2 (PMC13536644; doi:10.1186/s12889-026-29272-2)
Supplement: Supplementary file 1 [file 12889_2026_29272_MOESM1_ESM.pdf]

Focus Group Guides Used in the Study (English Translation)

I. Community Leaders

A. Introduction

1. Before the project began, were you aware of the public health problem related to dengue fever in your neighborhood?
    1.1. Please describe what the dengue situation was like in your neighborhood before the protective paint was applied.
2. Have you organized dengue prevention and control activities in your neighborhood?
    2.1. As a community, what activities against dengue have you carried out in your neighborhood?

B. Project Outreach

3. When you were invited to participate in the project, were the benefits clearly explained?
4. What motivated you to participate in this project?
5. What strategies did you use to motivate people and spread information about the project in your neighborhood?
6. Have you received any feedback from the University regarding the performance of the community promoters and painters?
    6.1. What characteristics did you consider when recommending them for this project?
7. Why do you consider it important to share the strategies being implemented in the community with the leaders?

C. Experiences and Evaluation of the Project

8. What was your experience like working in collaboration with the Dengue project coordinators?
9. What impact has the project had on your neighborhood?
10. For future interventions, what other communication strategies do you recommend to inform the entire community about the project?

D. Closing

11. Is there anything else you'd like to add that hasn't been discussed previously? Is there anything else you'd like us to discuss; any topic related to the project?

II. Health Promoters

A. Introduction

1. Have you had any experience working on health promotion programs? Please describe your experience.
2. What motivated you to participate in this project as a health promoter?

B. Training

3. Were the project objectives and work dynamics clearly explained and communicated? E.g., language used, time allotted, training content (consistent and aligned with the topic), trainer's mastery of the subject.
4. Were your roles and responsibilities in the project clearly explained to you?
5. Was the training sufficient to acquire the necessary knowledge and apply it during the project?
6. Do you have any suggestions or comments that might be helpful for improving future training sessions? E.g., audiovisuals, descriptive materials
7. What characteristics do you believe helped you become a good promoter?

C. Socialization phase: promotion of the insecticidal paint – door-to-door visits

8. Describe your experience promoting the protective paint.
9. Was the work schedule adequate for meeting daily goals?
10. Were the supplies (tools) provided to you adequate and sufficient for the proper performance of your duties?
    - 10.1. Were the questions on the field form easy to understand and fill out?
11. What strategies did you use to promote the protective paint and encourage household heads to use it? In other words, what did you do to convince the community? How did you speak to them? What did you say to persuade them?
12. Do you consider the flyer distributed to community members to have been a good strategy?
13. What difficulties and challenges did you face when promoting the protective paint? How did you address them?
14. What was the most rewarding part of your workday?
15. Do you think that implementing this strategy with people from the same community helped make people more receptive to your visit?
16. Did any incidents occur (that made you feel threatened or unsafe) during the course of the activity?
    - 16.1. Did you feel uncomfortable or intimidated by the household representative?
    - 16.2. Were you treated disrespectfully? What did you do about it?
    - 16.3. Were you verbally abused, or was the tone of voice used with you inappropriate? How did you handle the situation?
    - 16.4. Were you victims of workplace or gender discrimination? Did you report it to your supervisors?
17. Did you ever feel demotivated during the project implementation period?

D. Interpersonal and work relationships with the project team

18. How was your social relationship with your project team? Describe your experience.
    - 18.1. Did any disagreements arise within your work team that affected the completion of this project's activities?
    - 18.2. Did you have any conflicts with a coworker (including your supervisor and project coordinators) or did they disrespect you?
    - 18.3. Did the supervisor use clear and appropriate language when addressing you?
    - 18.4. Did you receive sufficient support and guidance from your team supervisor?
    - 18.5. What positive and negative aspects can you mention about your team supervisor?
19. Please describe situations where the project was affected or could have been affected. E.g., rain, police, power outages, COVID.

E. Project Evaluation

20. Do you have any suggestions for improving the project's work dynamics and becoming more efficient?

F. Closing

21. Is there anything you'd like to add that hasn't been discussed previously? Is there anything else you'd like us to discuss, such as a topic related to the project?

III. Painters – protective paint applicators

A. Introduction

1. Have you had any experience working in paint application and/or on health projects? Please describe your experience.
2. What motivated you to participate in this project as a healthcare painter?

B. Training

1. Were the project objectives and work dynamics clearly explained and communicated? E.g., language used, time allotted, training content (consistent and aligned with the subject matter), trainer's mastery of the subject.
2. Were your roles and responsibilities in the project clearly explained to you?
3. Was the training sufficient to acquire the necessary knowledge and apply it during the project?
4. Do you have any suggestions or comments that might be helpful for improving future training sessions? E.g., audiovisuals, descriptive materials
5. What characteristics do you believe helped you become a good painter?

C. Intervention phase: application of the insecticidal paint

3. What was your experience like participating in the Dengue project?
   3.1. What challenges and difficulties did you face?
4. Was the work schedule adequate for meeting daily goals?
5. Was the protective equipment provided adequate and sufficient for the job?
   5.1. Would you recommend the use of other safety equipment for future activities?
6. Did you find it uncomfortable to carry the protective paint and all the safety protective equipment you were given?
7. Was the paint application tool (paintbrush) adequate?
8. Did you experience any discomfort or symptoms during or after applying the insecticide paint?
   8.1. What kind of discomfort or symptoms did you experience?
9. Was your work affected by the health promoter you were assigned to work with as part of a team?
10. Do you think it was a good strategy to use the labels on the electricity meter as a guide to help you find the house?
11. Did any incidents occur (that made you feel threatened or unsafe) during the course of the activity?
12. *Question for women only*: Did you feel at a disadvantage in performing your job duties because you are women? Explain
13. Was the community receptive to your visit?
    13.1. Did you feel afraid when entering the house? Would you have preferred to go in groups of two?
    13.2. Did you feel uncomfortable or unsafe around the household representative?
    13.3. Were you disrespected? What did you do about it?
    13.4. Were you verbally abused, or was the tone of voice used with you inappropriate? How did you handle the situation?
    13.5. Were you victims of workplace or gender discrimination? Did you report it to your supervisors?
14. Did you ever feel demotivated during the project implementation period?
15. What positive feedback did you receive from the community?
16. Did you receive any negative feedback or complaints during or after applying the protective coating? Please list them.

D. Interpersonal and Work Relationships with the Project Team

17. How was your social relationship with your project team? Describe your experience.
    17.1. Did any disagreements arise within your work team that affected the completion of this project's activities?
    17.2. Did you have any conflicts with any of your teammates (including the supervisor and project coordinators) at work, or were you treated disrespectfully?
    17.3. Did the supervisor use clear and appropriate language when speaking to you?
    17.4. Did you receive sufficient support and guidance from your team supervisor?
    17.5. What positive and negative aspects can you mention about your team supervisor?
18. Please describe situations where the project was affected or could have been affected. E.g., rain, police, power outages, COVID.

E. Project Evaluation

19. Do you have any suggestions for improving the project's work dynamics and becoming more efficient?

F. Closing

20. Is there anything you'd like to add that hasn't been discussed previously? Is there anything else you'd like us to discuss, such as a topic related to the project?

IV. Vector control technicians

A. Training

1. Prior to the start of the project, you received training explaining the project's objective, logistics, features of the new vector control tool and how to use it, and safety measures to consider. Based on this:
2. Were the project objectives and work dynamics clearly explained and communicated? E.g., language used, time allotted, training content (consistent and aligned with the subject matter), trainer's mastery of the subject.
3. Were your roles and responsibilities in the project clearly explained to you?
4. Was the training sufficient to acquire the necessary knowledge and apply it during the project's implementation?
5. Do you have any suggestions or comments that might be helpful for improving future training sessions? E.g., audiovisual aids, descriptive materials

B. Fieldwork: application of insecticidal paint

6. Compared to other vector control methods you have used, what was your experience using insecticide paint as a new tool against the *Aedes* mosquito?
   6.1. What challenges or difficulties did you encounter when applying the insecticide paint? Please elaborate on your response
7. Was the work schedule adequate for meeting daily goals?
8. Did you find it uncomfortable to carry the insecticide paint and all the safety protective gear you were given?
9. Was the protective gear provided adequate and sufficient for the job?
   16.1. Would you recommend the use of other safety equipment for future interventions?
10. Did you experience any discomfort or symptoms during or after applying the insecticide paint?
    17.1. What kind of discomfort or symptoms did you experience?
11. How did you perceive community participation in implementing the insecticidal paint as a new vector control method, compared to other vector control methods you have used previously?
    11.1. What was the reaction (or what kind of reaction did they have or show) of the heads of households when the insecticidal paint was applied to their homes?
12. What techniques did you use to motivate and convince the community to accept the new vector control method (insecticidal paint)?
13. Please describe any difficulties or negative experiences you had with household heads during your visits.
14. What positive and negative feedback did you receive from the community after applying the insecticide paint?

C. Collaborative Work

In this project, you had the opportunity to work together with community members who served as health promoters and painters. Based on the above:

15. Describe your experience working with the community members who actively participated in the project's implementation (health promoters and painters).
16. Mention the positive aspects or advantages of working with the community.
17. What challenges or difficulties did you encounter while working with community members?
    17.1. Was the project affected, or could it have been affected?
18. Did the role of the health promoters facilitate acceptance of the insecticidal paint?
    18.1. Do you think this was due to the information they promoted or because they belong to the same neighborhood where they worked?

D. Project Evaluation

19. How would you evaluate the logistics and implementation of the project?
    19.1. What strategies would you recommend to improve the logistics of implementing the protective paint?
20. What are the positive and negative aspects of the project?
21. Do you consider protective paint to be a vector control tool that can be applied sustainably in the long term?
    21.1. What challenges do you think might arise in implementing the protective paint on a national scale?
22. Do you have any suggestions for improving the project's workflow and increasing efficiency?

E. Closing Remarks

23. Is there anything you'd like to add that hasn't been discussed previously? Is there anything else you'd like us to discuss, any topic related to the project?

V. Supervisors of promoters and painters

A. Project orientation

1. Did you have any previous experience supervising?
2. Were the project objectives and work dynamics clearly explained and communicated?
3. Were your roles and responsibilities in the project clearly explained to you?

B. On-site supervision

4. Describe your experience working on the Dengue project. How did you feel?
5. What strategies did you use to supervise your entire team?
6. What difficulties and challenges did you face? Explain in detail
   5.1. How did you address the problems?
7. Explain how the promoters and painters performed their duties
   6.1. Based on the promoter's duties, what shortcomings or negative aspects did you observe?
   6.2. Based on the painter's duties, what shortcomings or negative aspects did you observe?
   6.3. Did you observe any dishonest behavior from your work team (health promoters and painters)? What did you do as a supervisor to address the situation?
   6.4. What recommendations would you give us to prevent these types of situations from continuing to arise during the project? What strategies should we establish to ensure that these
7. Did you have any conflicts with any members of your work team, or were you ever disrespected? How did you handle the situation?
8. Did you feel unsafe or threatened in the neighborhoods where you worked? What did you do about it?
9. From your perspective, how was the community's reception?
   Did you notice any differences among the various neighborhoods where you worked?
10. What did you like most and least about working on this project?

C. Interpersonal and work relationships with the project team

11. How was your interaction or interpersonal relationship with your work team? Describe your experience.
    11.1. Did any disagreements arise within your work team that affected the completion of project activities?
    11.2. Did anyone on your work team (including other supervisors and coordinators) treat you disrespectfully?
    11.3. Did you receive sufficient support and guidance from the project coordinators?
12. Please describe situations where the project was affected or could have been affected by negative attitudes from a member of your work team.

D. Project Evaluation

13. How would you evaluate the logistics and execution of the project in terms of the dynamics between the promoter and the painter?
14. What are the positive and negative aspects of the project?
15. What suggestions can you offer to improve the project's workflow and increase efficiency?

E. Closing

16. Is there anything you'd like to add that hasn't been discussed previously? Is there anything else you'd like us to discuss, such as a topic related to the project?

VI. Community members

A. Introduction: Knowledge of the project

1. Through which media did you receive information about the project?

B. Outreach phase: visits by health promoters

2. Was the health promoter's preliminary door-to-door visit important? Why? Please elaborate on your answer
3. Were the individuals properly identified and wearing uniforms?
4. During the health promoter's visit, did you feel uncomfortable, threatened, and/or was the tone of voice they used disrespectful? Please elaborate
5. Were the instructions on how to prepare the laundry tub for painting explained to you in detail?
6. Did you find the information in the flyer you were given useful?
7. Was your participation in the Dengue project voluntary, or is there another reason unrelated to that?
   7.1. What motivated you to agree to participate in the project?
   7.2. What aspects of the protective paint caught your attention and led you to agree to have it applied to your laundry tank?

C. Intervention phase: application of the insecticidal paint

8. Were you satisfied with how the protective paint was applied to your laundry tank?
9. Did you feel more comfortable knowing that the promoters and painters were members of the community?

D. Experiences and Evaluation of the Protective Paint and the Project

10. Compared to other vector control methods you have used or continue to use (chlorine tablets, fish, etc.), what has your experience been or what is your opinion of the protective paint? Please elaborate on your answer.
    10.1. Have you noticed any changes in the tank (odor, color, taste, number of larvae and mosquitoes)?
    10.2. Have you or any member of your family noticed any changes in the use of water from the washing area after applying the protective paint?
11. Given the benefits of the protective paint that have been mentioned to you, would you purchase the protective paint if it were sold at an affordable price? Please elaborate on your answer.
12. Do you have any complaints about the protective paint and/or the project?
13. Do you know why some members of the neighborhood did not agree to participate in the project?
14. Do you feel that receiving the protective paint has brought any benefits to your home or community?
15. What recommendations can you offer to encourage more community members to paint their laundry room tanks with the protective paint?

F. Closing

16. Is there anything else you'd like to add that hasn't been discussed previously? Is there anything else you'd like us to discuss, such as a topic related to the project?
