# Supplementary material for: Community participation and operational lessons from implementing a protective coating as a novel vector control tool in dengue-endemic neighborhoods of Cúcuta and Los Patios, Colombia
Source: BMC Public Health. 2026 Sep 1;26:2505. doi: 10.1186/s12889-026-29272-2 (PMC13536644; doi:10.1186/s12889-026-29272-2)
Supplement: Supplementary file 2 [file 12889_2026_29272_MOESM2_ESM.pdf]

Appendix A. Operational details of the protective coating intervention

1. Volunteer roles and allocation

Each intervention area (approximately 2,000 households) was assigned an average of 10 pairs of volunteers (one promoter and one painter). One supervisor was assigned per cluster to oversee the work, ensure volunteer safety, and address operational challenges [39].

2. Scheduling and environmental conditions

Work was scheduled Monday to Saturday, from 7:30 a.m. to 12:30 p.m., when household members were more likely to be at home. Morning hours were preferred due to lower temperatures; afternoon temperatures could exceed 35°C, affecting volunteer performance [39].

3. First visit: promotion and household preparation

Promoters conducted house-to-house visits to explain the benefits of the protective coating, encourage participation, and provide instructions for preparing laundry tanks (emptying, cleaning, and drying for 24 hours). Flyers with project information, preparation steps, and contact details were distributed. Promoters visited households one day before painters [39].

4. Second visit: application of the protective coating

Painters applied the coating to eligible tanks and verified whether households had followed preparation instructions. Proper adherence required tanks to be empty, clean, and dry [39].

5. Household monitoring and documentation

A sticker with a unique code was placed on the electricity meter of each visited house. Stickers included the date of the visit, house code, and household decision (accepted, rejected, closed, not applicable). Painters also recorded whether the tank was protected with the coating [39].

6. Volunteer compensation

Community volunteers received a financial reward as appreciation for their time and to help cover transportation costs [39].
