# Supplementary material for: Community participation and operational lessons from implementing a protective coating as a novel vector control tool in dengue-endemic neighborhoods of Cúcuta and Los Patios, Colombia
Source: BMC Public Health. 2026 Sep 1;26:2505. doi: 10.1186/s12889-026-29272-2 (PMC13536644; doi:10.1186/s12889-026-29272-2)
Supplement: Supplementary file 3 [file 12889_2026_29272_MOESM3_ESM.pdf]

Appendix B. Fieldwork Tools, Equipment, and Documentation Procedures

Volunteers were supplied with a set of tools to document household data during their visit, such as a pencil, eraser, sharpie, clipboard, map of the work site, and a field form. Both the promoters and the painters registered the information of the visited houses on the same field form. The attempt to use cellphones for recording failed due to frequent robbery of these devices in the neighborhood.

The promoters recorded the date of the household visit, the house code, the type of laundry tank, the household's decision regarding acceptance of the paint, and any relevant observations. The painters, on the other hand, documented the date of the visit, and whether the laundry tanks of the households that accepted the paint were protected (painted with the PC), and additional observations, if necessary. Supervisors were responsible for collecting the forms filled by the promoters, and the following day, they handed these completed forms to the assigned partner painter to use them as a guide and visit only the households that had accepted the PC.

The tools provided to painters included the protective coating, face mask, googles, protective gloves, a 4" paint brush, and a container to place the brush in to facilitate the painting process. Volunteers reported that the tools provided were adequate for the effective execution of their tasks. The only inconvenience they pointed out was that during rainy days, they had to be more cautious to avoid getting the paper wet.

> "*I found it uncomfortable when it was raining, and my sheet got wet. I even bought a disposable plastic bag to keep the papers dry*". (Community volunteer from Los Patios).

The painters noted that the diameter of the paintbrush was larger than the paint container, which made it difficult to use when dipping the brush to load it with paint. They considered it appropriate to get a smaller brush or a wider container.

> "*The paint container was very narrow, so the paintbrush hairs would come out*". (Community volunteer from El Trigal).

On the other hand, some painters claimed to feel uncomfortable using safety equipment (goggles, face masks, and gloves) while painting, as they had difficulty breathing and painting while wearing a disposable face mask and protective gloves.

> "*It was a little uncomfortable for me wearing gloves, but I tried my best to do my work*". (Community volunteer from El Trigal).

> "*The materials were of good quality, but I would have preferred latex gloves or the long dishwashing gloves, as my hands got covered in paint while I was painting*".
